# Aloe Vera-Mediated Te Nanostructures: Highly Potent Antibacterial Agents and Moderated Anticancer Effects

**DOI:** 10.3390/nano11020514

**Published:** 2021-02-18

**Authors:** David Medina-Cruz, Ada Vernet-Crua, Ebrahim Mostafavi, María Ujué González, Lidia Martínez, A-Andrew D. Jones III, Matthew Kusper, Eduardo Sotelo, Ming Gao, Luke D. Geoffrion, Veer Shah, Grégory Guisbiers, Jorge L. Cholula-Díaz, Christelle Guillermier, Fouzia Khanom, Yves Huttel, José Miguel García-Martín, Thomas J. Webster

**Affiliations:** 1 Department of Chemical Engineering, Northeastern University, Boston, MA 02115, USA; vernetcrua.a@northeastern.edu (A.V.-C.); ebi.mostafavi@gmail.com (E.M.); a.jones@northeastern.edu (A.-A.D.J.III); gao.min@northeastern.edu (M.G.); shah.vee@northeastern.edu (V.S.); websterthomas02@gmail.com (T.J.W.); 2 Nanomedicine Science and Technology Center, Northeastern University, Boston, MA 02115, USA; 3 Stanford Cardiovascular Institute, Stanford, CA 94305, USA; 4 Department of Medicine, Stanford University School of Medicine, Stanford, CA 94305, USA; 5 Instituto de Micro y Nanotecnología, IMN-CNM, CSIC (CEI UAM+CSIC), Isaac Newton 8, 28760 Tres Cantos, Spain; maria-ujue.gonzalez@csic.es; 6 Materials Science Factory, Instituto de Ciencia de Materiales de Madrid, ICMM-CSIC, Sor Juana Inés de la Cruz 3, 28049 Madrid, Spain; lidia.martinez@icmm.csic.es (L.M.); huttel@icmm.csic.es (Y.H.); 7 Department of Physics and Astronomy, University of Arkansas at Little Rock, 2801 South University Avenue, Little Rock, AR 72204, USA; mikusper@ualr.edu (M.K.); ldgeoffrion@ualr.edu (L.D.G.); gxguisbiers@ualr.edu (G.G.); 8 School of Engineering and Sciences, Tecnologico de Monterrey, Av. Eugenio Garza Sada 2501, Monterrey, NL 64849, Mexico; esr_97@outlook.com (E.S.); jorgeluis.cholula@tec.mx (J.L.C.-D.); 9 Carl Zeiss SMT, Inc., PCS Integration Center, Peabody, MA 01960, USA; christelle.guillermier@zeiss.com (C.G.); fouzia.khanom@yahoo.com (F.K.)

**Keywords:** nanoparticles, aloe vera, tellurium, biomedical, antibacterial, anticancer

## Abstract

Cancer and antimicrobial resistance to antibiotics are two of the most worrying healthcare concerns that humanity is facing nowadays. Some of the most promising solutions for these healthcare problems may come from nanomedicine. While the traditional synthesis of nanomaterials is often accompanied by drawbacks such as high cost or the production of toxic by-products, green nanotechnology has been presented as a suitable solution to overcome such challenges. In this work, an approach for the synthesis of tellurium (Te) nanostructures in aqueous media has been developed using aloe vera (AV) extracts as a unique reducing and capping agent. Te-based nanoparticles (AV-TeNPs), with sizes between 20 and 60 nm, were characterized in terms of physicochemical properties and tested for potential biomedical applications. A significant decay in bacterial growth after 24 h was achieved for both Methicillin-resistant *Staphylococcus aureus* and multidrug-resistant *Escherichia coli* at a relative low concentration of 5 µg/mL, while there was no cytotoxicity towards human dermal fibroblasts after 3 days of treatment. AV-TeNPs also showed anticancer properties up to 72 h within a range of concentrations between 5 and 100 µg/mL. Consequently, here, we present a novel and green approach to produce Te-based nanostructures with potential biomedical applications, especially for antibacterial and anticancer applications.

## 1. Introduction

Antimicrobial resistance to antibiotics (AMR) has emerged as a threat to the healthcare system due to the misuse and overuse of antibiotics over the past century. Data from the Centers for Disease Control and Prevention (CDC) indicate that at least 2 million people become infected with antibiotic-resistant bacteria in the United States annually and about 23,000 die; even worse, this is expected to exponentially grow in the upcoming years [1,2,3]. Simultaneously, cancer causes around 600,000 patients to die every year just in the United States [4]. Although the use of radiotherapy and chemotherapy has improved life expectancy, decreasing the number of deaths over the last few years by 27%, they are not free of side effects and other drawbacks [5,6]. Even more, tumor cell chemotherapy-drug resistance is a rising concern, often now leading to total chemotherapeutic treatment failure [7].

Consequently, there is a strong need for new treatments for both AMR and cancer using strategies that do not induce bacterial resistance and chemo/radiotherapy resistance, respectively. Nanotechnology provides an alternative solution with the implementation of nanomaterials in medicine [8]. Nanomaterials are highly reactive due to their high-surface-to-volume-ratios and may show novel properties compared to the bulk materials. Moreover, a vast number of synthetic methodologies have been reported, allowing for the effortless production of nanoparticles (NPs) with different biomedical features. For instance, NPs can decrease bacterial survival rates without being highly toxic to mammalian cells [9] or show a dose-dependent anticancer effect towards some of the deadliest tumors [10]. So far, extensive research has been conducted concerning the use of different materials to produce effective NPs including pure metals, such as silver (Ag) [11] or gold (Au) [12], to metal oxides, like titanium oxide (TiO_2_) [13] and zinc oxide (ZnO) [14] or non-metals and metalloids, such as selenium (Se) [15,16] and tellurium (Te) [17,18,19].

In particular, Te is considered a rare and mildly toxic metalloid, belonging to the chalcogen family [20]. This metalloid has been widely used in industry, especially in solar cell technology, photoconductors, and thermoelectric devices [21,22]. Still, applications of the Te are limited due to the high price and geographical restriction for its production. However, due to the generation of the metalloid as a by-product of copper refining, as well as its dependence on certain technologies, prices for Te can be amongst the most volatile of any element. For instance, between 2009 and 2016, Te prices ranged from US$ 30 per kilogram to as high as $450 per kilogram [23]. Since then, prices have drifted downward, and by early 2016, Te prices hit their lowest levels in over a decade, which are projected to continue dropping due to higher efficiencies in the extraction of Te from slime. As such, Te supply should be adequate to support business alternatives well into the future and to broaden the availability of the metalloid in different geographical areas [24]. Therefore, with prices nowadays compared to other metals, and due to a consequent increasing interest in research synthesis alternatives to the element, many different approaches have been tested over the years. However, no extensive research has been completed for the use of Te in biomedical applications, with only a few studies on the use of Te-based nanomaterials as unique antibacterial and anticancer agents. For instance, citric-mediated tellurium nanoparticles (TeNPs) showed potential anticancer activity towards melanoma cells, while biologically synthesized tellurium nanorods (TeNRs) slowed bacterial proliferation [25]. Some of the advantages of these Te-based nanomaterials compared to others materials reported in literature, such as Ag or Au, for biomedical applications are related to: (a) different mechanisms of antimicrobial efficacy linked to increased levels of oxidative stress that do not support bacteria resistance as can be found in Ag-nanomaterials [26] and (b) a specific chemistry of Te similar to sulfur that allows for its incorporation into amino acids, such as cysteine and methionine, hence becoming useful as a biological marker [27].

TeNPs can be synthesized using different approaches, from physical methods, such as laser ablation [28], to chemical means, like precipitation [29]. However, some of these traditional approaches often have drawbacks related to the use of high temperature and pressure, acidic pH medium, harsh chemicals, and, most importantly, the production of toxic by-products, which are a severe threat to the environment. Common issues related to NP aggregation may also occur depending on NP synthesis rate, which is a severe limitation for biomedical applications [30]. Furthermore, these NPs usually require some form of functionalization using different organic groups, polymers, or alternative structures to avoid aggregation and reduce toxicity towards human cells [31,32].

To overcome some of the aforementioned limitations, green chemistry principles have been combined with nanotechnology to give rise to a new field termed “green nanotechnology” [33]. One of the most successful green-nanotechnological approaches, also called biogenic or biosynthetic synthesis, comes from the use of living organisms, such as bacteria, fungi, or plants, to obtain nanomaterials [34]. Despite extensive research with other elements, there are a limited number of studies related to the biogenic synthesis of TeNPs [35,36,37].

Along these lines, aloe vera (AV) has been used as a therapeutic agent since ancient times. The AV gel has been employed for the treatment of several skin cuts, burn abnormalities, constipation, colic, skin diseases, and worm infestation [38,39,40]. The AV gel or mucilage consists of 99.3% water, while the remaining 0.7% is made up of solids with a high content of glucose and mannose [41,42]. This provides AV with wound and burn healing properties as well as anti-inflammatory activity, UV protection, antiarthritic properties, as well as reported antibacterial effects, due to the presence of many biologically active constituents, such as lignin, hemicellulose, or pectins [43]. Furthermore, many other different phytoconstituents such as vitamins, minerals, sugars, anthraquinones, saponins, salicylic acid, and amino acids can be found in the extracts [44]. Due to their low cost and environment-friendly nature coupled with their chemical reducing properties, the AV extract was selected here as a reducing and stabilizing agent to prepare different metallic nanomaterials [45,46,47]. Once the ions are reduced and start to nucleate, enzymes and proteins are weakly bonded and act as a complexing agent, stabilizing the nanostructures and avoiding aggregation due to surface charge.

To the best of the authors’ knowledge, this is the first report on the synthesis of microwave-assisted green TeNPs using AV extracts. Here, the Te nanostructures were further purified and characterized in terms of surface chemistry, composition, and morphology. Finally, they were tested for their antibacterial and anticancer properties as well as cytotoxicity towards healthy human cells at low to medium concentrations.

## 2. Materials and Methods

### 2.1. Synthesis and Purification of AV-TeNPs

The precursors employed for the green synthesis of aloe vera-mediated tellurium nanoparticles (AV-TeNPs) included sodium tellurite (Na_2_TeO_3_) (Sigma Aldrich, St. Louis. MO, USA) and an AV extract obtained from leaves that were purchased from a local vendor (Boston, MA, USA) and sterilized (by means of a wash with hot water and ethanol) to remove potential contaminants. For the extract preparation, 100 g of AV leaves were cut into small pieces and boiled with 100 mL of deionized (DI) water for 30 min. After boiling, the solution was cooled and filtered using a 0.2 µm pore size sterilization filter (Fisher, Cambridge, MA, USA) connected to a vacuum line. The cooled brownish leaf broth was then stored in a refrigerator at 4 °C prior to use in experiments. For the AV-TeNPs preparation, 20 mL of a concentration of 2 mM Na_2_TeO_3_ was mixed with the same volume of AV extract. The mixture was introduced in a microwave and heated at 350 W for 10 s, after which it was allowed to cool down at room temperature. After the formation of a black suspension, it was centrifuged at 10,000 rpm for 20 min. The pellet was collected and washed twice with DI water to remove potential unreacted precursors. After both washes, the pellet was lyophilized, and the final powder was collected for further experiments. Then, the diluted samples were prepared by mixing fixed amounts of DI/autoclaved water and portions of the powder to generate liquid samples with desired concentrations. 

### 2.2. High-Resolution Transmission Electron Microscopy Analysis

TEM analysis was done using high-resolution transmission electron microscopy (HR-TEM) (Hitachi, Clarksburg, MD, USA). For the analysis, one drop of the diluted sample was placed on a 200 mesh Cu grid coated with a layer of carbon 20 ± 5 µm in thickness. The dried sample was placed into a JEOL double tilt specimen holder and inserted into a JEOL 2100-F TEM operating at 200 kV. The sample was allowed to stabilize at a column pressure of 9.0 × 10^−6^ Pa in the microscope before imaging. The absence of formvar as a grid support increases the transmission of the electron beam. Therefore, a condenser lens aperture with a diameter of 50 µm was chosen to reduce unnecessary electron interaction with the sample, employing an emission current of 119 µA. The microscope was aligned by performing JEOL’s standard alignment procedures, followed by high-resolution alignment. The images were collected using a GATAN camera (Gatan Inc., Pleasanton, CA, USA) and processed with GATAN digital micrograph software (Gatan Microscopy Suite 3, Pleasanton, CA, USA).

### 2.3. X-ray Photoelectron Spectroscopy Analysis

For X-ray photoelectron spectroscopy (XPS) characterization, drops of a solution of diluted AV-TeNPs were deposited onto a clean conductive copper substrate. After water evaporation, the sample was loaded in a vacuum load-lock chamber and then transferred to an ultrahigh vacuum XPS system (SPECS, Berlin, Germany). The XPS chamber had a base pressure of 10^−10^ mbar and was equipped with a hemispherical electron energy analyzer (SPECS Phoibos 100 spectrometer) and an Al Kα (1486.29 eV) X-ray source. The angle between the hemispherical analyzer and the plane of the surface was kept at 60°. Broad scan spectra were recorded using an energy step of 0.5 eV and a pass-energy of 40 eV while specific core level spectra (Te 3d, O 1s, and C 1s) were recorded using an energy step of 0.1 eV and a pass-energy of 20 eV. Data processing was performed with CasaXPS software (version 2.3.13Dev30, Casa Software Ltd., Cheshire, UK). The absolute binding energies of the photoelectron spectra were determined with reference to the C 1s core level at 285 eV [48,49]. The contribution of the Al Kα satellite lines were subtracted.

### 2.4. Fourier-Transform Infrared and Raman Spectroscopy

Structural analysis of the Te-based nanostructures was completed by infrared spectroscopy using a Fourier-transform infrared (FT-IR) spectrometer, PerkinElmer Spectrum 400 FT-IR, FT-NIR (PerkinElmer Inc., Waltham, MA, USA) in attenuated total reflectance (ATR) mode. The sample for FT-IR analysis was prepared using 5 µg of AV-TeNPs placed on the sample holder. The FT-IR spectra were acquired using a wavenumber range of 500–4000 cm^−1^ with a resolution of 4 cm^−1^. The spectrum was normalized, and the baseline was corrected using Spectrum™ software (6.3.4, PerkinElmer, Waltham, MA, USA). For Raman spectroscopy, one drop of the sample was placed on a Si wafer manufactured by the Monsanto electronic materials company, with a thickness of 200 ± 20 µm and a resistance of 30 ± 10 Ω. The silicon (Si) wafer was then placed in an enclosed container in order for the sample droplet to dry overnight. The Si wafer was initially cleaned by sonication at 35 kHz. This was done while being submerged in acetone, followed by ethanol, then DI water, and finally DI water again, sonicated in each solvent for 5 min. Raman spectroscopy analysis was performed using EZRaman-N from Enwave Optronics, Inc. (Irvine, CA, USA)with a wavelength of 532 nm at 500 mW.

### 2.5. Bacterial Culture Preparation and Antibacterial Analysis

Strains of both Gram-negative and Gram-positive bacteria were used in this study to determine the antibacterial activity of the AV-TeNPs. Methicillin-resistant *Staphylococcus aureus* (MRSA) (ATCC 4330; ATCC, Manassas, VA, USA) and multidrug-resistant *Escherichia coli* (MDR *E. coli*) (ATCC BAA-2471; ATCC, Manassas, VA, USA) bacteria were used and cultured following ATCC instructions. Prior to inoculation, the bacterial cultures were maintained on agar plates at 4 °C. Bacteria were introduced into 6 mL of sterile Luria-Bertani (LB) (bioPLUS, bioWORLD) medium in a 15-mL Falcon centrifuge tube and incubated at 37 °C and 200 rpm for 24 h. The optical density (OD) of the bacterial cultures was measured at 600 nm using a spectrophotometer (SpectraMax M3, Molecular Devices, Sunnyvale, CA, USA). The bacterial suspension was then diluted to a concentration of 10^6^ colony-forming units per milliliter (CFU/mL) and stored at 4 °C until use. Growth curves and other bacterial analyses were performed using a plate reader with a SpectraMax^®^ Paradigm^®^ Multi-Mode Detection Platform.

For the antimicrobial assay, 100 μL of AV-TeNPs in LB medium at different concentrations were mixed with 100 μL of bacteria in LB medium and then added to each well of a 96-well plate (Thermo Fisher Scientific, Waltham, MA, USA). For the untreated controls, 100 μL of bacteria were mixed with 100 μL of LB medium without NPs. The final volume per each well was 200 µL. Once the plate was prepared, the absorbance of all samples was measured at 600 nm on an absorbance plate reader every 2 min for 24 h with no shaking. Negative controls containing only NPs and media were used to determine the absorbance caused by the NPs.

Colony counting assays were also performed as follows: bacteria were seeded in a 96-well plate and treated with different concentrations of NPs for 8 h inside an incubator at 37 °C. Then, the 96-well plate was removed from the incubator, and all the samples were diluted with phosphate buffer saline (PBS) in a series of vials to either ×100, ×1000, or ×10,000. Three drops from a 10 µL aliquot of each dilution were then placed in an LB-Agar plate and incubated for 8 h inside the incubator at 37 °C. The resulting number of colonies formed in each plate was counted at the end of the incubation time.

### 2.6. In Vitro Cytotoxicity Assay with Biogenic AV-TeNPs

#### 2.6.1. In Vitro Cell Culture

Primary human dermal fibroblast (HDF) cells (Lonza, CC-2509, AMP, Hopkinton, MA, USA) and melanoma cells (ATCC CRL-1619, Manassas, VA, USA) were cultured at 37 °C and 5% CO_2_ in a humidified atmosphere in Dulbecco’s Modified Eagle Medium (DMEM; Thermo Fisher Scientific, Waltham, MA, USA), supplemented with 10% fetal bovine serum (FBS; ATCC 30-2020™, American Type Culture Collection, Manassas, VA, USA) and 1% penicillin/streptomycin (Thermo Fisher Scientific). When the cells were 70% confluent, they were trypsinized and seeded into 96-well tissue culture plates (Thermo Fisher Scientific). HDF and melanoma cells were seeded at a final concentration of 5 × 10^4^ cells per well in 100 µL of cell medium. The seeded well plates were kept in a humidified atmosphere at 37 °C and 5% CO_2_ for cell viability and metabolic studies as described below.

#### 2.6.2. Metabolic Activity of the Cells Using MTS Assays

Specifically, (3-(4,5-Dimethylthiazol-2-yl)-5-(3-carboxymethoxyphenyl)-2-(4-sulfophenyl)-2H tetrazolium) (MTS) assays (CellTiter 96^®^ Aqueous One Solution Cell Proliferation Assay, Promega, Madison, WI, USA) were used to determine cellular metabolic activity to assess cytotoxicity. After being seeded, cells were incubated for a period of 24 h at 37 °C in a humidified incubator with 5% CO_2_. Then, the culture medium was replaced with 100 µL of fresh cell medium containing various concentrations ranging from 25 to 175 µg/mL of biogenic AV-TeNPs. Prior to in vitro assessment, NPs were sterilized through UV exposure for 30 min. Cells were cultured for an extra 24 h in the same conditions, followed by washing the cells with PBS and replacing the medium with 100 µL of the MTS solution (prepared using a mixing ratio of 1:5 of MTS:medium). After the addition of the solution, the 96-well plate was incubated for 4 h to allow for a color change. Then, the absorbance was measured at 490 nm on an absorbance plate reader (SpectraMAX M3, Molecular Devices, San Jose, CA, USA) for cell viability after exposure to the AV-TeNP concentration. Cell viability was calculated by dividing the average absorbance obtained for each sample by the one obtained for the control sample and then multiplied by 100. Controls containing either cells and media or just media were also included in the 96-well plate to identify the average growth of cells without nanoparticles and to determine the absorbance of the media. Cell experiments were carried out twice, one for just 24 h and another for a total time of 48 h.

#### 2.6.3. Metabolic Activity of the Cells Using PrestoBlue Assays

The metabolic activity of the cells was determined using a PrestoBlue cell viability kit (Thermo Fisher Scientific, Cambridge, MA, USA) following the manufacturer’s protocol. In brief, the PrestoBlue solution was added to the media at days 1, 3, and 5 post-seeding to constitute 10% of the whole media in the wells. Following this, the cells were incubated for 45 min at 37 °C. Fluorescence intensity of the solutions was recorded using a microplate reader (Bio-Tek Inc., Winooski, VT, USA) at 590–615 nm emission and 535–560 nm excitation.

#### 2.6.4. Cell Viability Using Live/Dead Assays

A live/dead assay kit (Invitrogen, Carlsbad, CA, USA) was used to determine the viability of the cells attached to the well plates containing AV-TeNPs for 1, 3, and 5 days post-seeding following the manufacturer’s instructions. Briefly, the attached cells in the wells were stained with ethidium homodimer-1 (EthD-1, 2 μL/mL in PBS) for dead cells and calcein-AM (0.5 μL/mL in PBS) for live cells. Following this, the cells were incubated for 15 min at 37 °C, then the stain was removed, and the cells were washed with PBS three times. Finally, the stained cells were imaged using an inverted fluorescence microscope (Zeiss Axio Observer Z1, Carl Zeiss microscopy, Thornwood, NY, USA), and images were analyzed using Image J software (ImageJ 1.49 g, LOCI, University of Wisconsin, WI, USA). The cell viability percentage was calculated by dividing the number of live dead cells by the total number of cells.

### 2.7. Reactive Oxygen Species (ROS) Analysis

For ROS quantification, 2′,7′-dichlorodihydrofluorescein diacetate (H2DCFDA) was used. Human melanoma cells were seeded in a 96-well plate at a concentration of 5 × 10^4^ cells/mL in DMEM medium and in the presence of different concentrations of AV-TeNPs as well as in control medium without any nanoparticles. The cells were cultured under standard culture conditions (37 °C in a humidified incubator with a 5% CO_2_ atmosphere) for 24 h before the experiment. Briefly, the ROS indicator was reconstituted in anhydrous dimethyl sulfoxide (DMSO) to make a concentrated stock solution that was kept and sealed. The cell growth medium was then carefully removed, and a fixed volume of the indicator in PBS was added to each one of the wells at a final concentration of 10 μM. The cells were incubated for 30 min at an optimal temperature, and the loading buffer was removed thereafter. Fresh medium was added and the cells were allowed to recover for a short time. Positive controls were included stimulating the oxidative activity with hydrogen peroxide to a final concentration of 50 μM. The baseline for the fluorescence intensity of a sample of the loaded cell culture period of exposure was determined by subtracting the values of the controls. The intensity of fluorescence was then observed by flow cytometry. Measurements were taken using a fluorescence wavelength at 530 nm when the sample was excited at 485 nm. Fluorescence was also determined for the negative control (untreated loaded with dyed cells maintained in a buffer).

### 2.8. Statistical Analysis

All experiments were repeated in triplicate (N = 3) unless otherwise indicated to ensure the reliability of the results. Statistical significance was assessed using Student’s *t*-tests, with an alpha value less than 0.05 being statistically significant. Results were displayed as the mean ± standard deviation (Prism 9 software). Further, statistical analysis using Hedges’ g mean difference was performed on the growth parameters of the bacterial species. Antibiotic effectiveness, half-maximal effective concentration (EC_50_) (the concentration of NPs that induced a response halfway between the baseline and maximum after a specified exposure time), and the minimum inhibitory concentration (MIC) (the lowest concentration of NPs that will inhibit the visible growth of the bacteria) were calculated following modeling methods that are explained in the results and discussion section. 

## 3. Results and Discussion

### 3.1. Synthesis and Purification of AV-TeNPs

AV-TeNPs were successfully synthesized following a simple and straightforward protocol using an AV extract to reduce tellurite (TeO_3_^2−^) ions dissolved in an aqueous media to elemental tellurium (Te^0^) in the form of NPs. Some of the biologically active components present in the AV extract are vitamins, enzymes, minerals, sugars, lignin, saponins, and several amino acids, which represent a standard composition for many other plant extracts. Sugars derived from the mucilage layer of the plant, known as mucopolysaccharides [42,50], are responsible for the ionic reduction [51]. The presence of a free aldehyde group or an open ketone group within the structure of the mucopolysaccharide allows them to reduce metallic ions. Once the metallic ions have been reduced, NPs are formed from small metallic nuclei, which tend to naturally arrange themselves via a process called “Ostwald ripening” [52]. Subsequent stabilization of the AV-TeNPs was hypothesized to be caused by: (a) the presence and action of the same sugars that lead to reduction [53] or (b) the functionality of other organic molecules, such as fatty acids (cholesterol, campesterol, beta-sitosterol, and lupeol) [54], with a high presence in the extracts [42,47]. It was hypothesized that the selective interaction of these organic compounds with the forming NP nuclei led to the specific crystallographic shapes present in the nanostructures. 

### 3.2. High-Resolution Transmission Electron Microscopy Analysis 

**Figure 1:**
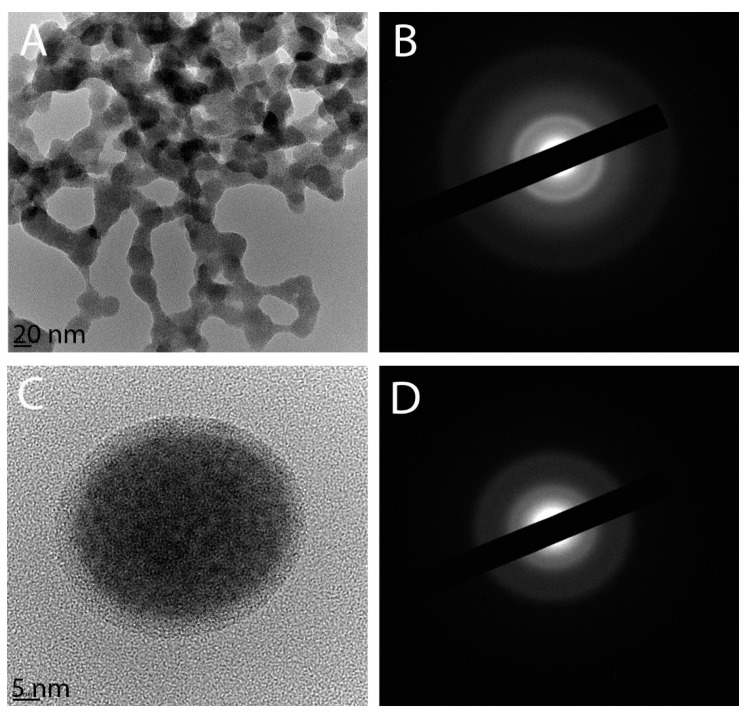
TEM images of aloe vera-mediated tellurium nanoparticles (AV-TeNPs): agglomerates (**A**), a single spherical Te-based nanostructure (HRTEM) (**C**), and their corresponding diffraction patterns (**B**,**D** for **A**,**C**, respectively).

The AV-TeNPs were characterized using TEM to observe the size and morphology of the structures (Figure 1). Small spherical NPs were found to be aggregated in clusters of different extensions, with a size distribution between 20 and 60 nm, which was found using particle size distribution experiments (based on at least 100 NPs) (Figure 1A). These clusters were easily disrupted, and small aggregates were pulled apart after a few minutes of sonication, indicating a weak interaction between the NPs (Figure 1C).

**Figure A1:**
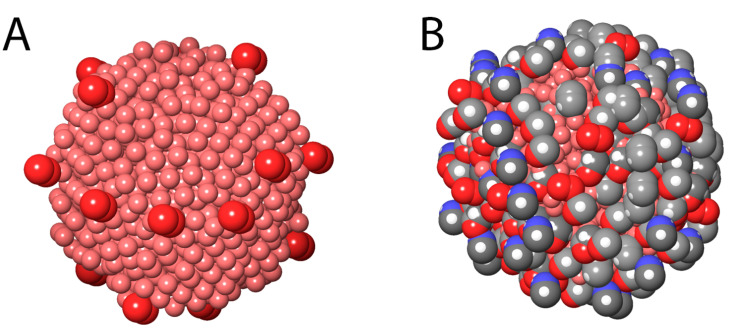
Schematic illustration of the models of aloe vera-mediated tellurium nanoparticles (AV-TeNPs) without a biomolecule-based external layer/coating (**A**, represented with pink and some red spheres) and with a biomolecule-based external layer/coating (**B**, represented by gray, red, blue, and white spheres) obtained from the simulations. The nanoparticles are shown as a space-filling model (CPK model). Tellurium (Te) is in pink, carbon (C) in gray, oxygen (O) in red, nitrogen (N) in blue, and hydrogen (H) in white. Both dark and light gray represent carbon atoms, and the color difference is because of the light source of the simulation software.

**Figure A3:**
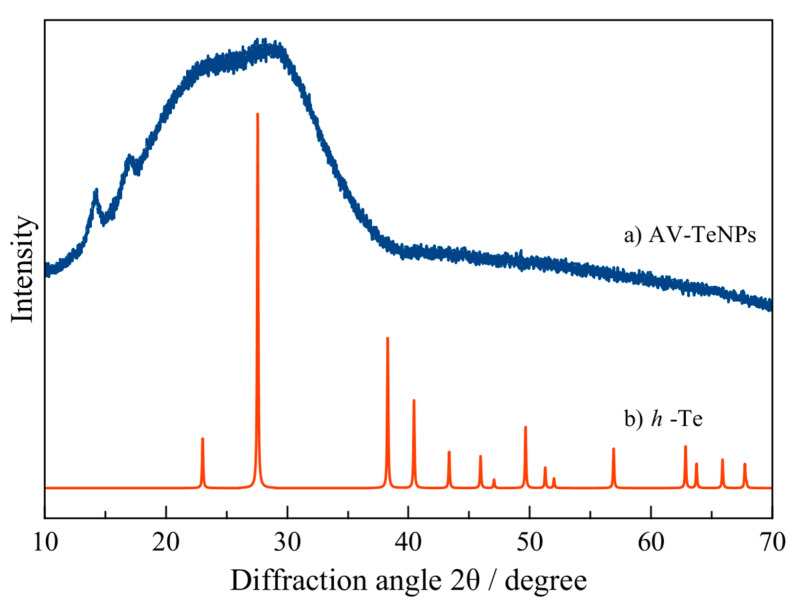
XRD analysis of the AV-TeNPs.

From the TEM imaging, a coating surrounding the NPs was observed (Figure 1A,C). FTIR analysis (*vide infra*) confirmed that the composition of the coating was related to the AV extract employed as the raw material. The AV-TeNPs were amorphous, as can be seen from the electron diffraction patterns in Figure 1B,D. This amorphous structure can be explained from a thermodynamic point of view. Indeed, the amorphous-trigonal phase transition of tellurium is known to be size-dependent at the nanoscale. Guisbiers et al. [17] predicted the amorphous-trigonal transition of TeNPs to occur at 45 °C for a 50 nm size particle. The amorphous patterns obtained by electron diffraction agree with the X-ray diffraction analysis, see Appendix (Figure A3), as well as with the atomistic simulations (Appendix A.1 and Figure A1). Moreover, the Z-potential value of the AV-TeNPs colloid was −24 ± 2 mV pointing out the relative stability of the system in water (see Appendix A.2, in the Appendix, for further stability analyses of the colloids).

### 3.3. Fourier-Transform Infrared and Raman Spectroscopy Analysis

**Figure 2:**
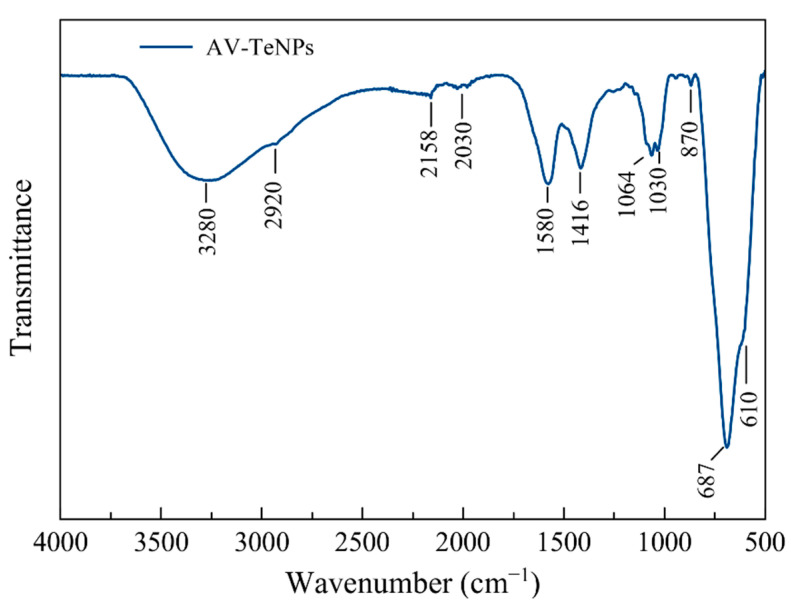
FT-IR spectrum of the aloe vera-mediated TeNPs (AV-TeNPs).

Further structural analysis of the AV-TeNPs was carried out by FT-IR spectroscopy. Most of the bands observed in the FT-IR spectrum (Figure 2 and Table 1) correspond to the functional groups of the most representative phytochemical constituents found in the AV extract, including polysaccharides (e.g., acemannam, galactan, and pectin), proteins, vitamins, enzymes, organic acids, phenolic substances, phytosterol, flavones, organic acids, and quinones [55,56,57]. The broad band at 3280 cm^−1^ is assigned to the stretching mode of the –OH group from alcohols and phenols. The small vibrational band at 2920 cm^−1^ may be assigned to the symmetrical and asymmetrical C-H stretching of aliphatic –CH and –CH_2_ groups. The small signals observed at around 2158 and 2030 cm^−1^ may corresponds to the alkynyl C=C stretch in the AV extract [58]. The bands at 1580 and 1416 cm^−1^ are characteristic of C=C from the aromatic rings and symmetrical –COO stretching vibrations, respectively. The vibrational bands in the region of 1060–1030 cm^−1^ may be attributed to the presence of C–O and C–N stretching vibrations of rhamnogalacturonan, a side-chain constituent of pectins and aliphatic amines, respectively. The small band at 870 cm^−1^ may be related to the C-H out-of-plane deformation of monosaccharides [56,57]. Finally, the strong vibrational band at 687 cm^−1^ and the shoulder at 610 cm^−1^ are due to the symmetrical and asymmetrical axial Te-O stretching vibrations [59,60]. These results demonstrate the presence of organic materials that can be ascribed to the phytochemicals from the AV extract, which act as capping agents of the Te-based NPs.

**Figure 3:**
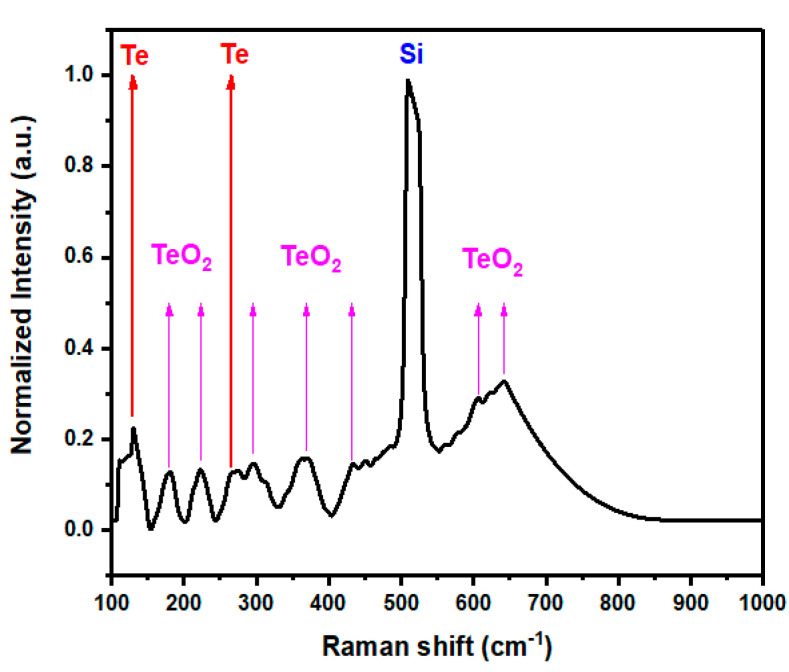
Raman spectra of the AV-TeNPs deposited on top of a silicon wafer. The peaks of Te and TeO_2_ are clearly identified.

The Raman analysis (Figure 3) allowed one to observe a characteristic band at 520 cm^−1^, which corresponds to the Si substrate onto which the sample (AV-TeNPs) was deposited. The bands at 132 cm^−1^ (E-bond stretching mode of Te) and 266 cm^−1^ (related to the second-order harmonic of the E vibrational mode of Te) correspond to pure Te, hence demonstrating the presence of metallic Te. On the other hand, the bands at 180, 224, 296, 368, 434, 606, and 642 cm^−1^ correspond to γ-TeO_2_ [62,63].

### 3.4. Chemical Composition Studies of the AV-TeNPs

**Figure 4:**
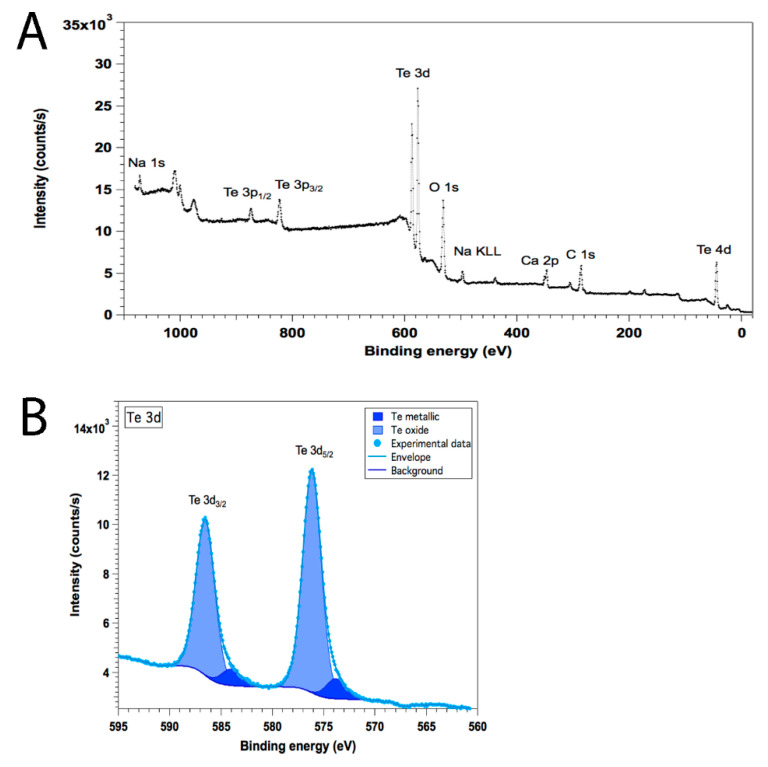
Broad scan XPS spectrum (**A**) and Te 3d XPS core level spectrum with the corresponding metallic and oxide components (**B**).

The chemical composition of the sample was extracted from the broad scan XPS spectrum displayed in Figure 4A. The resulting concentrations of the different elements are presented in Table 2.

Different elements besides Te were found in the sample. Principally, O and C were associated with the organic coating coming from the AV extract. Moreover, significant amounts of Ca and Na were detected in the sample. These elements are naturally present in high quantities in AV. Surprisingly, no relevant presence of nitrogen could be measured at the N 1s core level that remained within the experimental noise. This is in opposition with other green-synthesized Te nanostructures where N was detected [64]. The analysis of the Te 3d core-level spectrum (Figure 4B) revealed the presence of a small Te^0^ component that represented between 4% and 5% of the Te, and a strong oxide component. This is not surprising since as XPS is mostly sensitive to the surface, the signal comes mainly from the outer shell of the NPs. The metallic and oxide Te 3d_5/2_ components were found at 573.8 and 576.1 eV, respectively, and their full width at half maximum (FWHM) were similar (only 4% variation), which guarantees that none of the components overestimated the other. These binding energies are slightly higher than expected (573.0 and 576.0 eV) [65,66], indicating that charging effects on these TeNPs are stronger than in other compounds [19].

A detailed explanation of the XPS analysis for the rest of the elements can be found in Appendix A.5. Moreover, Appendix A.4 and Appendix A.6 contain the results of scanning electron microscopy coupled with energy-dispersive X-ray spectroscopy (SEM-EDX) and secondary ion mass spectrometry Imaging (SIMS), respectively. They confirm the existence of the organic coating with C and O and small quantities of the other elements.

### 3.5. Determining the Antimicrobial Activity of AV-TeNPs

**Figure 5:**
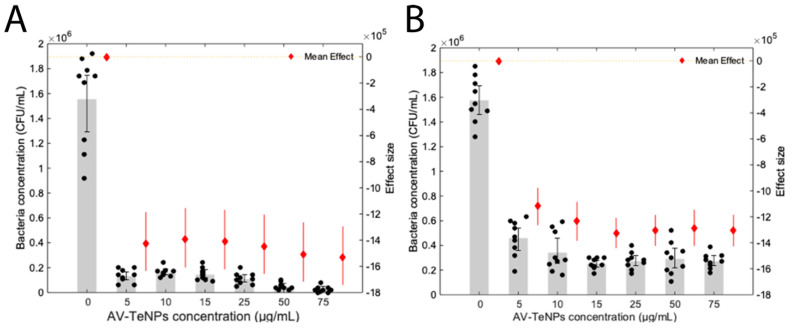
Colony counting assays of (**A**) multidrug-resistant (MDR) *E. coli* and (**B**) methicillin-resistant *Staphylococcus aureus* (MRSA) treated with AV-TeNPs; N = 3, where 3 measurements were taken for each biological replicate (denoted as a black bold dot, ●). The magnitude of the mean of the technical and biological replicates and the standard error are shown in the bar chart with error bars. All results showed a *p*-value < 0.01 versus control. Each mean effect size (denoted as a diamond ♦) with a 95% confidence interval is indicated by the ends of the vertical error bars plotted on the right axis.

Bacterial growth studies in the presence of AV-TeNPs were carried out for both Gram-negative and Gram-positive drug-resistant bacteria. Colony counting unit assays were conducted using MDR *E. coli* (Figure 5A) and MRSA (Figure 5B) confirming a dose-dependent inhibition behavior of the Te-based nanostructures over a range of 5–75 µg/mL for MDR *E. coli* and 0–15 µg/mL for MRSA. The nanostructures were effective (*p*-value < 0.01) towards both bacterial strains at a range of concentrations between 5 and 75 µg/mL. The strength of the AV-TeNPs effect can be quantified using the effect size, i.e., the difference between the control and trial means normalized by the standard deviation. The mean of the effective size was −14 ± 0.55 × 10^5^ against MDR *E. coli* and −13 ± 0.77 × 10^5^ against MRSA across all concentrations, showing a stronger effect against MDR *E. coli*. The MIC and EC_50_ values were calculated and presented in Table 3 to quantify the static and dynamic antibacterial effects of the AV-TeNPs, respectively.

These values showed a substantial decrease in the MIC for the AV-TeNPs compared to other biogenic NPs found in the literature. For example, the antibacterial effect of TeNPs produced by *Stenotrophomonas maltophilia* SeITE02 and *Ochrobactrum* sp. MPV1 was investigated, which were tested against *Staphylococcus aureus* and *E. coli* with MIC values of 1000 and 500 µg/mL, respectively [35]. *E. coli* can possess a resistance against tellurite, in a MIC range of 2–1024 µg/mL with resistant genes being present both in common laboratory strains and strongly associated with virulent O157:H7 lineages, although not directly associated with Te exposure [67,68]. 

Similarly, the EC_50_ values against MDR *E. coli* and MRSA were much lower than those found by Zonaro et al. of 160 and 150 µg/mL for TeNPs against *E. coli* and for *S. aureus*, respectively [35]. Puzyn et al. developed a theoretical, quantitative structure–activity relationship for computing the EC_50_ value of metal oxide-derived NPs based on the enthalpy of formation of the oxide from the pure metal. Whether due to the production of ROS or metabolic interruption, tellurite is hypothesized to be the primary agonist against *E. coli* [69,70], so it is appropriate to use it as a model to estimate the EC_50_ for TeNPs at a concentration of 36 µg/mL. This value falls in the range of those found in literature and again is higher than that for our AV-TeNPs. 

**Figure A8:**
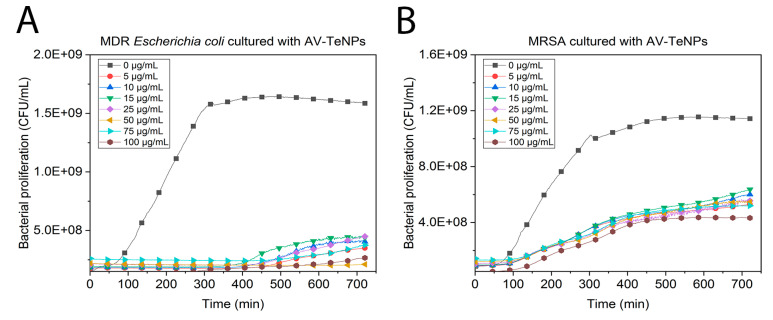
Effect of AV-TeNPs on the growth of a 1 × 10^6^ CFU/mL suspension of multidrug-resistant (MDR) *E. coli* (**A**) and methicillin-resistant *Staphylococcus aureus* (MRSA) (**B**) for 24 h in the presence of different concentrations of AV-TeNPs. The values represent the mean ± standard deviation.

**Figure A9:**
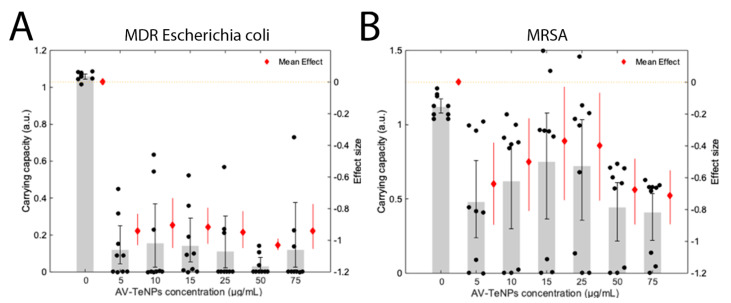
The carrying capacity and effect size against the shared control 0 concentration are shown in the above Cumming estimation plots for MDR *Escherichia coli* (**A**) and MRSA (**B**) experiments. Each mean difference is depicted as a diamond with a 95% confidence interval indicated by the ends of the vertical error bars. Three measurements were taken for each biological replicate (denoted as a black bold dot, ●).

**Figure A10:**
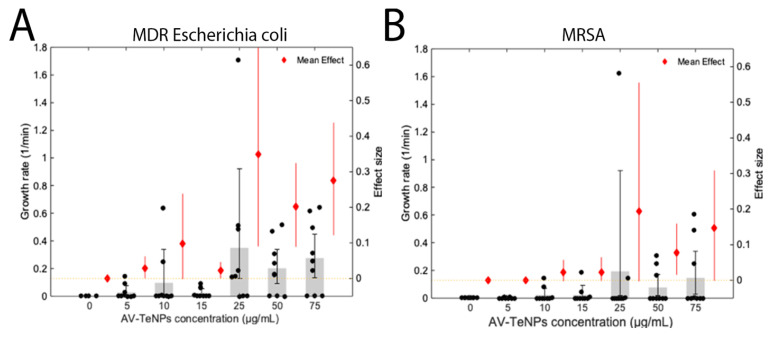
The growth rate and effect size against the shared control 0 concentration are shown in the above Cumming estimation plots for MDR *Escherichia coli* (**A**) and MRSA (**B**) experiments. Each mean difference is depicted as a diamond with 95% confidence interval indicated by the ends of the vertical error bars. Three measurements were taken for each biological replicate (denoted as a black bold dot, ●).

**Figure A11:**
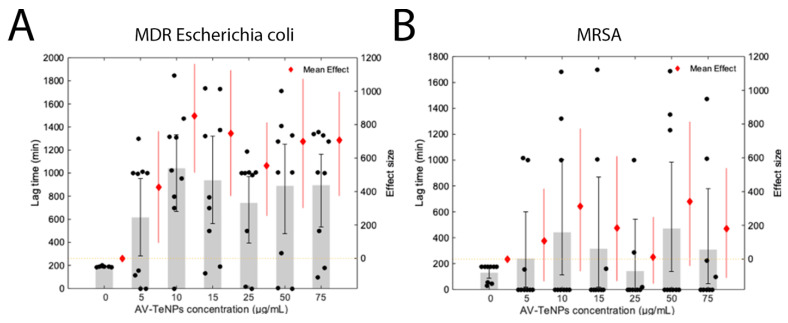
The lag time and effect size against the shared control 0 concentration are shown in the above Cumming estimation plots for MDR *Escherichia coli* (**A**) and MRSA (**B**) experiments. Each mean difference is depicted as a diamond with 95% confidence interval indicated by the ends of the vertical error bars. Three measurements were taken for each biological replicate (denoted as a black bold dot, ●).

The growth parameters were also analyzed to investigate the mechanism of action of AV-TeNPs, as proposed by Brauner et al. [70], see Appendix A.7 and Figure A8, Figure A9, Figure A10 and Figure A11. These figures showed a slight dose-dependent effect of MDR *E. coli* to the AV-TeNPs and a dose-independent response by MRSA. It is hypothesized that a stronger electrostatic attraction decreases the concentration required to have an antibacterial response. As the Z-potential for the AV-TeNPs is −24 mV, the NPs are more “positive” than *E. coli* (*Z* = −30 mV), and so, they are efficiently attached to them, whereas those AV-TeNPs are more “negative” than *S. aureus* (*Z* = −14 mV), and they are not efficiently attached [71,72]. Therefore, the effect of AV-mediated synthesis is likely due to surface modification. The largest effects were observed with carrying capacity, with no statistical significance on the growth rate showing that the mechanism of AV-TeNP action likely does not come during cell division and will subsequently be more difficult to develop a resistance against it; however, further study is needed.

### 3.6. Reactive Oxygen Species (ROS) Analysis 

**Figure A12:**
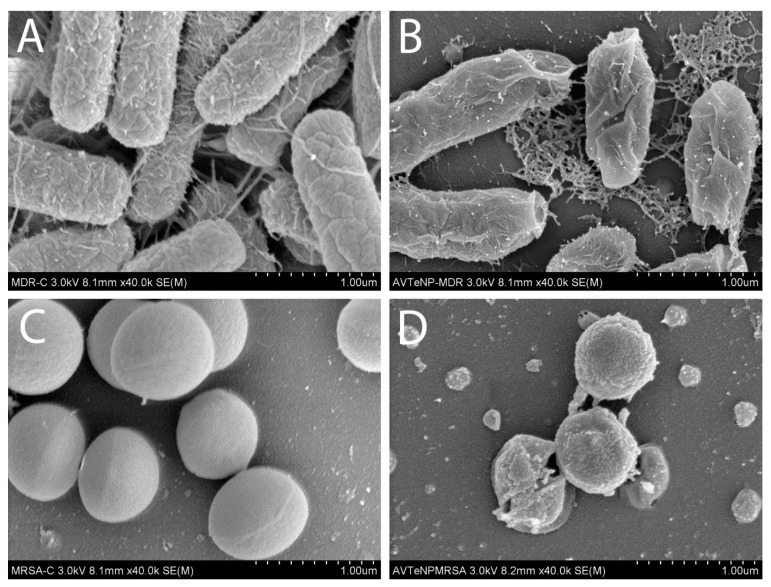
Representative SEM micrographs of multidrug-resistant (MDR) *E. coli* and methicillin-resistant *Staphylococcus aureus* (MRSA) (**A**,**C**, respectively) without and with AV-TeNP treatment (**B**,**D**, respectively).

The SEM study of the interaction between bacteria and NPs showed that the treatment with the AV-TeNPs induced disruption of the outer cell membrane and cell lysis (Section A.8 and Figure A12). This cell membrane damage can be attributed to ROS since these species can partially or totally destroy lipids, nucleic acids, and proteins, which contributes to the physiology of the cells. We have, therefore, analyzed the production of ROS in the presence of AV-TeNPs.

**Figure 6:**
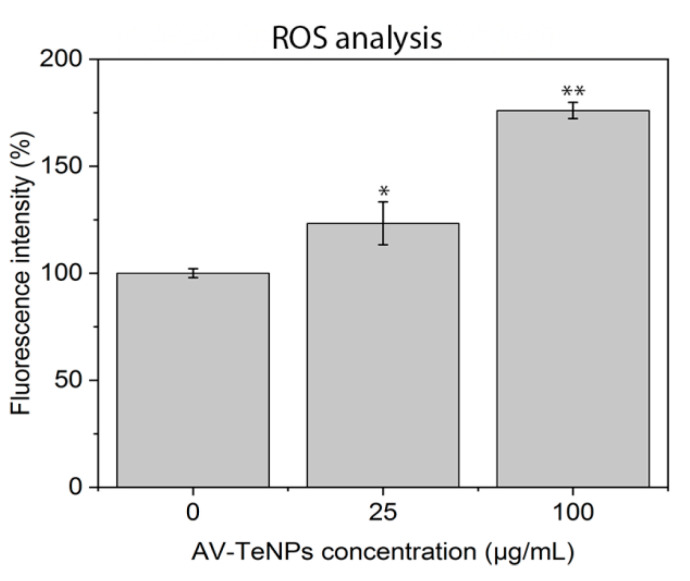
Reactive oxygen species (ROS) induced by AV-TeNPs in human melanoma cells experiments. A trend of the release of the species with the increase in AV-TeNPs concentration for the same timeframe is seen. N = 3. Data is represented as mean ± SD; * *p* < 0.05, ** *p* < 0.01 (compared to 0 concentration).

**Figure A13:**
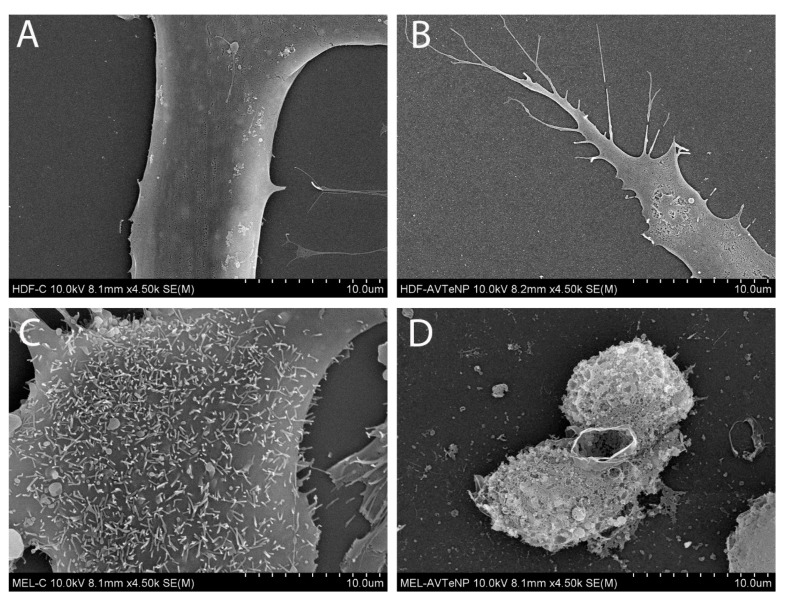
SEM micrographs of control human dermal fibroblast (HDF) and melanoma cells (**A**,**C**, respectively) without and with AV-TeNP treatment (**B**,**D**, respectively).

ROS analysis was performed by exposing human melanoma cells to different concentrations of AV-TeNPs: 25 and 100 µg/mL. The cells were in contact with the AV-TeNPs for a period of 24 h. Therefore, the ROS could be quantified in the cell media. The results (Figure 6) showed an increase in ROS production when AV-TeNPs were present in the media, with a dose-dependent effect. An excess level of ROS in the cell surroundings might lead to substantial damage to proteins, nucleic acids, lipids, membranes, and organelles within the cell. Besides, at sufficient doses, powerful oxidants cause severe damage to macromolecules all over the membranes and cytoplasm [73]. This can lead to cell death by apoptosis, as has been found after SEM analysis of the melanoma cells in the presence of TeNPs (Appendix A.8 and Figure A13). Cell apoptosis is distinguished by cell shrinking, membrane blebbing, chromatin condensation, and nuclear fragmentation, followed by the formation of apoptotic bodies [74]. From these results, we can hypothesize that the increase in ROS production is related to the detected antimicrobial activity of AV-TeNPs as well as with toxicity mechanisms towards human cells. It is relevant to mention here that the anticancer effect of the nanoparticles (to be presented below) can also be attributed to the generation of ROS [18].

### 3.7. In vitro Cytotoxicity Assay with AV-TeNPs

**Figure 7:**
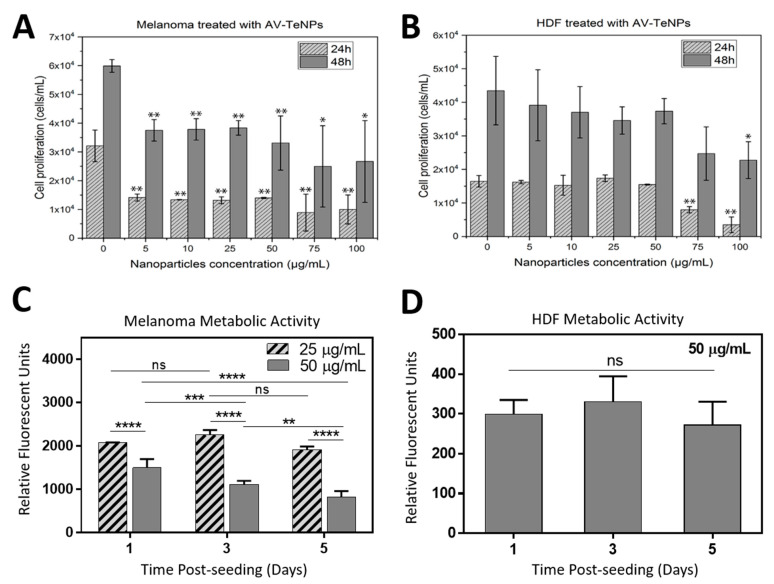
Melanoma (**A**) and human dermal fibroblast (HDF) (**B**) cells in the presence of AV-TeNPs at concentrations ranging from 5 to 100 μg/mL. N = 3. Data is represented as mean ± SD; * *p* < 0.05, ** *p* < 0.01 compared to the control. (**C**) Melanoma cells treated with AV-TeNPs at two different concentrations of 25 and 50 µg/mL. (**D**) Metabolic activity of HDF cells treated with AV-TeNPs at 50 µg/mL. Asterisks mark significance levels of *p* < 0.01 (**), *p* < 0.001 (***), and *p* < 0.0001 (****), ns = nonsignificant, and n = 5.

To establish a complete assessment of the cytotoxicity of the AV-TeNPs towards both cancer and healthy human cells, cell proliferation in the presence of several concentrations of NPs was analyzed. A dose-relative cell proliferation decay was found when the NPs were cultured with HDF cells after 1 and 2 days of incubation (Figure 7B). Specifically, a low cytotoxic effect was found in a range of concentrations between 5 and 50 µg/mL at 24 and 48 h. Therefore, the optimal working range for the AV-TeNPs (where the NPs could be considered biocompatible) was determined to be between 5 and 50 µg/mL in experiments up to 48 h. Moreover, a substantial decay in melanoma cell proliferation was found when the AV-TeNPs were present in the media for the complete range of concentrations (Figure 7A). Over 24 h, all concentrations led to a similar inhibition of cell proliferation, while at 48 h, a similar behavior was found with a significant statistical difference compared to the control.

Based on the abovementioned results, AV-TeNPs with concentrations of 25 and 50 µg/mL were selected to investigate the metabolic activity and growth of the HDF and melanoma cells over a longer period of incubation up to 5 days. Figure 7C demonstrates the metabolic activity of melanoma cells treated with AV-TeNPs with two different concentrations of 25 and 50 µg/mL obtained from the PrestoBlue assay. The experimental results revealed that AV-TeNPs at a concentration of 25 µg/mL were not significantly effective in killing melanoma cells, while 50 µg/mL AV-TeNPs were strongly effective in killing the cancer cells. These results suggest that the anticancer properties of the NPs are concentration dependent, and in fact, there would be a critical concentration of the NPs with the capability to kill the melanoma cells. Furthermore, we evaluated the effect of AV-TeNPs on HDF cells to show how toxic these NPs could be when implanted onto the skin. For this purpose, a 50 µg/mL concentration of AV-TeNPs was selected, which was able to kill most of the melanoma cells. Figure 7C,D revealed that while the 50 µg/mL AV-TeNPs did not promote melanoma cell growth, the HDF cells grew in the presence of the NPs.

**Figure 8:**
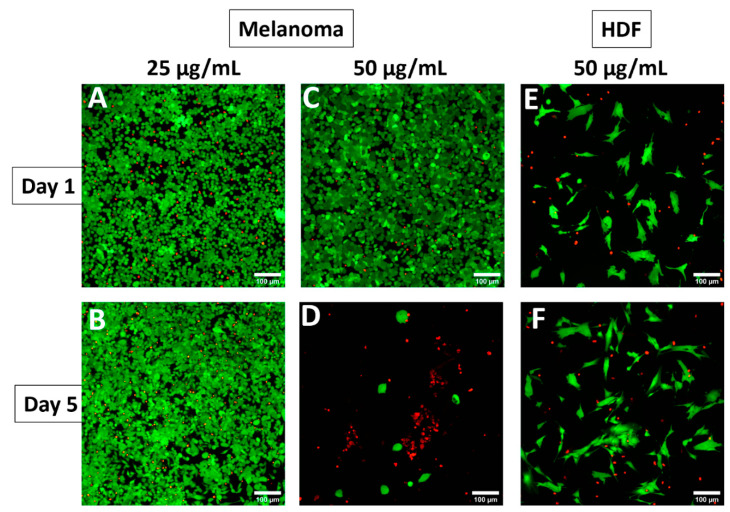
In vitro cytocompatibility study via live/dead assays. Representative live/dead images of melanoma cells in the presence of (**A**,**B**) AV-TeNPs at a concentration of 25 µg/mL at days 1 and 5 post-seeding onto well plate and (**C**,**D**) AV-TeNPs at a concentration of 50 µg/mL show significant anticancer properties of the NPs and (**E**,**F**) representative live/dead images of HDF cells in the presence of AV-TeNPs at a concentration of 50 µg/mL at days 1 and 5 post-seeding onto well plates.

The anticancer activity of AV-TeNPs with two different concentrations of 25 and 50 µg/mL was assessed in more detail by seeding melanoma cells onto the NPs and evaluating the cell viability using a commercial live/dead kit on days 1, 3, and 5 post-seeding. The results revealed that melanoma cells were growing fine in the presence of the lower concentrations of AV-TeNPs (i.e., 25 µg/mL), as shown in Figure 8A,B, while at higher concentrations, the NPs significantly inhibited the growth of the melanoma cancer cells (Figure 8C,D), confirming the results obtained from the metabolic activity. As can be seen in Figure 8A–D, the viability of the melanoma cells at day 1 of culture is more than 90%, and in the case of the AV-TeNP concentration of 25 µg/mL, it remains almost the same during 5 days, while for the 50 µg/mL AV-TeNPs dose, the viability dramatically drops over 5 days of culture. This corroborates the significant anticancer property of these AV-TeNPs at the 50 µg/mL concentration. Interestingly, the authors found that although the HDF cells were not proliferating following exponential growth in the presence of the NPs during 5 days of culture, they remained at the same level of viability (Figure 8E,F), which can confirm the cytocompatibility of these AV-TeNPs (at a 50 µg/mL concentration) to normal non-cancerous skin cells.

The half-maximal inhibitory concentration (IC_50_) values were calculated and presented in Table 4, with the aim to study the potency of the AV-TeNPs to inhibit the growth of both HDF and melanoma cells. 

These IC_50_ values differ from others found in literature, showing a decrease in the IC_50_ values for the AV-TeNPs. For example, Yang et al. investigated the anticancer effect of Te nanodots synthesized using hollow albumin nanocages, that were tested against 4T1 tumor cells with an IC_50_ value of 880 µg/mL [75]. The SEM study of the interaction between human cells and the NPs showed that the HDF cells were able to successfully proliferate in the presence of the NPs, with no apparent disruption or alteration of membranes or healthy growth (Figure A13). On the other hand, the presence of the AV-TeNPs induced a severe presence of bubbles and membrane disruption within the melanoma cell population, and characteristic morphologies were found indicating apoptotic mechanisms of cell death. As has been previously discussed, an increase in the NP concentration triggers a rise in the production of ROS (Figure 6). Therefore, this behavior might be related to the concentration of ROS within the cellular media.

The results presented here indicated that the NPs might undergo some internalization process into the cells, most likely related to pino- and macropinocytosis [76], as those processes have been usually reported for NPs of similar sizes, followed by interactions with cellular membrane receptors and internalization via endocytosis [77]. An important factor to consider for the cellular uptake of the AV-TeNPs is surface charge [78]. Although positively charged NPs have higher internalization than negatively charged NPs, the uptake of the former may disrupt the integrity of the cellular membrane and lead to an increase in toxicity and cell death, which was not observed in cell structure characterization [79]. Once the NPs reach the interior of the cells, intracellular trafficking might have been impacted by endosomes, with some of them escaping and being released into the cytoplasm [80], or autophagy, leading to a potential accumulation in the perinuclear region [81]. Furthermore, the AV-TeNPs might have suffered from elimination and excretion processes by the cells. Similarly, most types of NPs suffer from particle dissolution, lysosomal exocytosis, or exosome secretion as the main mechanisms for NP cellular excretion. If used in vivo, the elimination routes of the AV-TeNPs would rely on their intrinsic biodegradability or small size to be removed renally or by means of hepatobiliary elimination via transcytosis through hepatocytes in the liver, resulting in transport into the biliary system, then into the gastrointestinal tract, and eventual elimination via feces [82]. 

## 4. Conclusions

This research reported the plant-based green generation of TeNPs using aloe vera extracts. The AV-TeNPs have a spherical shape, exhibiting a core with metallic Te surrounded by a shell with oxidized tellurium and an organic coating composed of phytochemical components from the AV extract. The antimicrobial activity of the TeNPs was studied, showing a powerful inhibition of bacterial proliferation when tested towards antibiotic-resistant Gram-negative and Gram-positive bacteria for a range of NPs concentrations between 5 and 50 µg mL^−1^. Fibroblast viability in the presence of the AV-TeNPs was reported with no significant cytotoxicity in the same concentration range, while a moderated dose-dependent anticancer effect against melanoma cells was demonstrated.

## 5. Patents

The patent application PCT/US 2020/012436 entitled Tellurium Nanostructures with Antimicrobial and Anticancer Properties Synthesized by Aloe Vera-Mediated Green Chemistry was prepared and submitted by the coauthors from Northeastern University using some of the content of this article.

## Figures and Tables

**Table 1:** FT-IR main features of aloe vera-mediated tellurium nanoparticles (AV-TeNPs).

Wavenumber (cm^−1^)	Vibrational Modes	References
3280	*-OH (phenols and alcohols)*	[58]
2920	*-CH & -CH_2_ (aliphatic groups)*	[57]
2160–2030	*alkynyl C=C stretch*	[58,59]
1580	*C=C (aromatic rings)*	[58]
1416	*-COO- (carboxylate groups)*	[57]
1060	*C-O (rhamnogalacturonan)*	[57]
1030	*C-N (aliphatic amines)*	[58]
870	*C-H (monosaccharides)*	[57]
687–610	*Te-O*	[60,61]

**Table 2:** Composition of the sample extracted from the broad energy range XPS spectrum.

Technique	C %_at_	O %_at_	Te %_at_	Ca %_at_	Na %_at_
XPS	36.4	35.8	20	4.3	3.5

**Table 3:** Half-maximal effective concentration (EC_50_) and minimum inhibitory concentration (MIC) values for AV-TeNPs against multidrug-resistant (MDR) *E. coli* and methicillin-resistant *Staphylococcus aureus* (MRSA).

Drug-Resistant Bacterium	EC_50_ (µg/mL)	MIC (µg/mL)
MDR *E. coli*	1.9	3.53
MRSA	2.4	11.61

**Table 4:** Half-maximal inhibitory concentration (IC_50_) values (µg/mL) for the AV-TeNPs cultured with HDF and melanoma cells.

Exposed Cells	24 h	48 h
HDF	70.89	71.35
Melanoma	2.761	4.050

**Figure A2:**
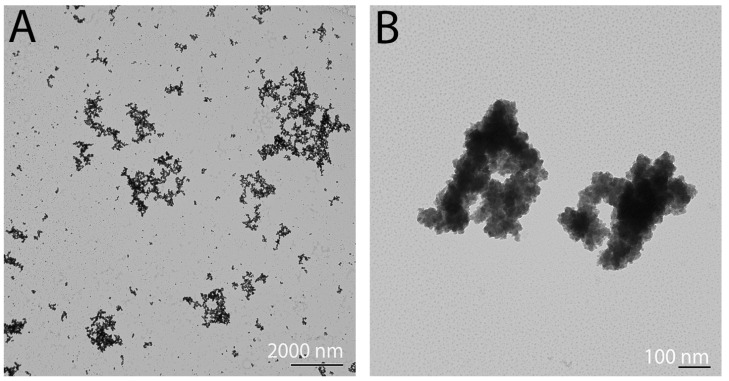
Transmission electron microscopy (TEM) characterization of 60 days old AV-TeNPs at low (**A**) ad high (**B**) magnification.

**Figure A4:**
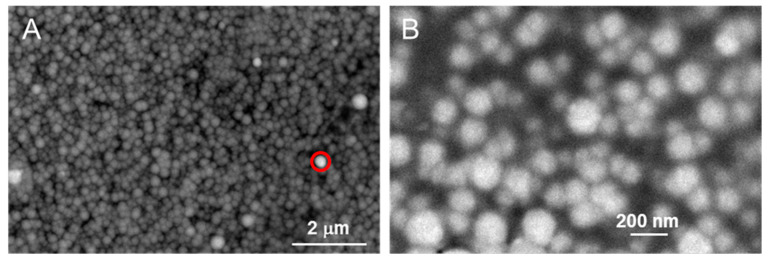
Scanning electron microscopy (SEM) characterization of AV-TeNPs at low (**A**) and high (**B**) magnifications. The red circle in (**A**) indicates the location of a spot for EDX analysis (see text for details).

**Figure A5:**
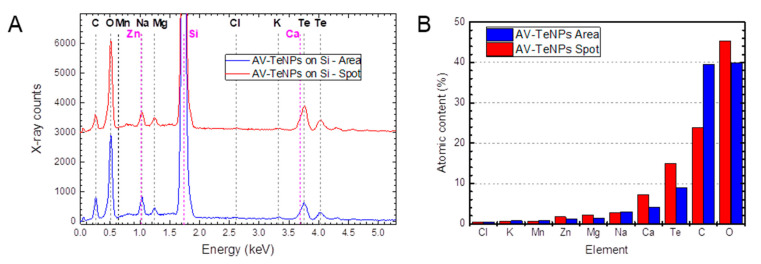
Energy-dispersive X-ray spectroscopy (EDX) characterization of the AV-TeNPs showing the presence of Te within the sample, as well as other elements corresponding to the reactants. (**A**) X-ray spectra, taken in different zones: spot corresponds to the big TeNP surrounded by the red circle in Figure A4A, and the area corresponds to the whole same image. The elements corresponding to the detected peaks are indicated in the graph. (**B**) Quantification of the elements is obtained from the spectra. As can be observed, the Te concentration is higher on top of the observed NP, corroborating their assignment as TeNPs.

**Figure A6:**
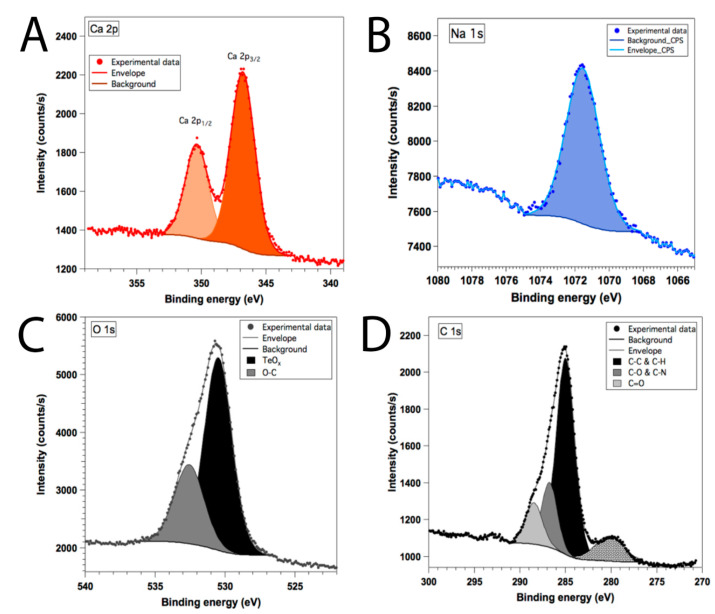
Ca 2p XPS core level spectrum (**A**), Na 1s XPS core level spectrum (**B**), O 1s XPS core level spectrum and the corresponding components used in the fitting procedure (**C**), and C 1s XPS core level spectrum and the corresponding components used in the fitting procedure (**D**).

**Figure A7:**
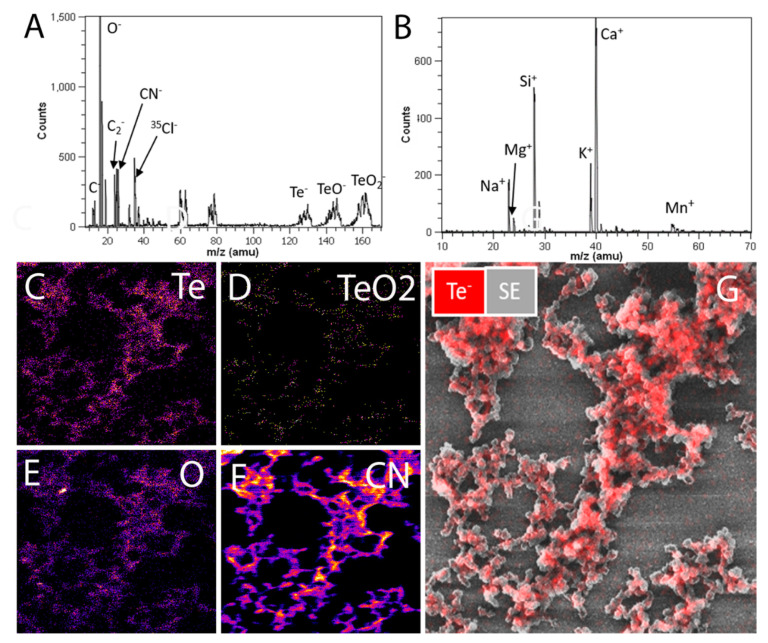
Secondary ions mass spectra acquired from a 50 µm × 50 µm field of view with a 20 keV energy neon ion beam: (**A**) positive mode and (**B**) negative mode; 4 µm × 4 µm field of view SIMS images of aggregated AV-TeNPs: SIMS images of Te, TeO_2_, O. and CN negative secondary ions acquired simultaneously with a 4 pA current neon beam at 20 keV of energy (**C**–**F**) and an overlay of the co-registered in situ SE (gray) and Te ions (red) images (**G**).

## Data Availability

The data presented in this study are available upon reasonable request to the corresponding authors.

## Appendix A

### Appendix A.1. Schematic Modeling of the AV-TeNPs

To have a representative view of the NPs at the atomic level, a schematic illustration model of the nanosystem was completed using the Schrödinger Materials Science 2019-1 suite with the information gathered from the physical characterization of the structures. AV-TeNPs were modeled at the atomic level. Figure A1 shows the models of AV-TeNPs without (A) and with a coating (B) modeled at the atomic level with all the details regarding morphology. The coating is referred to as the organic external layer made of biomolecules and fragments of those related to the raw materials, in this case, AV extracts. The systems, which contain 1050 atoms and 2480 atoms, respectively, for A and B, were fabricated by arranging atoms/molecules into a desired geometry shape and volume with data obtained from the modeling software based on physical and chemical characterization. The diameters of the AV-TeNPs are 50.21 and 62.13 nm, respectively, close to that in our experimental observations. The schematic illustration explains the experimental findings and discloses aspects that cannot be scrutinized by experiments. It is worth pinpointing that O is mainly at the surface of the NPs, and the amount of O increases with the organic coating (external layer). In this section, we have presented two detailed models of AV-TeNPs: coated and uncoated at the atomic level. These models are also able to run simulations in further studies, such as molecular dynamics simulation to determine the stability of this model, interaction with mammalian cell membranes or bacterial membrane bilayers to better understand the mechanism of antibacterial activity, and more.

### Appendix A.2. Stability Analysis

To verify the stability of the Te-based nanostructures, TEM imaging on the samples after 60 days of synthesis were carried out (Figure A2). In general, it is evident that the samples kept their original morphologies and features. For instance, the 60-day-old AV-TeNPs sample is composed of partially agglomerated Te nanostructures, as seen in Figure A2B.

Stability analysis was further confirmed through Z-potential measurements of the freshly synthesized and 60-day-old AV-TeNPs colloids (stored at room temperature in a glass vial). In general, a colloid or suspension is considered fully stable if the Z-potential is above a critical value of ±30 mV, whereas it is almost fully collapsed in large clusters if the value is below ±5 mV. Given the measured Z-potential values for the colloids (fresh and 60-day-old samples, see Table A1), they can be considered moderately stable since the values are close to −30 mV, although a slight decrease in value may be due to the aggregation found in the 60-day-old AV-TeNPs samples.

**Table A1:** Z-potential values for fresh and 60-day-old aloe vera-mediated tellurium nanoparticles (AV-TeNPs). The pH of the colloids was 7.0 ± 0.2.

	Z-potential (mV)	
Nanostructure	As-synthesized	60 days old
AV-TeNPs	−24 ± 2	−17 ± 3

### Appendix A.3. X-ray Diffraction Analysis

The X-ray diffraction pattern was recorded using a Rigaku MiniFlex 600 operating with a voltage of 40 kV, a current of 15 mA, and Cu-Kα radiation (wavelength = 1.542 Å). The measurement was done at room temperature with a step width of 0.005° (2θ) and a scan speed of 0.25 °/min. The sample for the XRD analysis was prepared by drop-casting 5 mL of the diluted AV-TeNPs onto the sample holder.

In Figure A3, the experimental XRD pattern of the AV-TeNPs sample was compared with the calculated XRD pattern of elemental hexagonal tellurium (*h*-Te) [83]. The experimental XRD pattern showed a standard profile of an amorphous material, i.e., a very broad hump at relatively low diffraction angles (2θ = 10–40°) without any distinct peaks [84]. The diffraction peaks at 2θ = 14.2° and 17.0° could not be assigned to any crystallographic phase related to an expected Te-based compound. However, it can be ascribed to small crystallites of unknown composition. In summary, the analysis of the sample indicates an amorphous structure of Te in agreement with TEM results.

### Appendix A.4. Scanning Electron Microscopy Analysis

A field-emission scanning electron microscope (FE-SEM), model FEI Verios 460 (FEI Europe B.V., Eindhoven, Netherlands), using selective secondary/backscattered electrons detection was used for additional morphological characterization. The subsequent observation was done using 10 µL of diluted AV-TeNPs that were deposited on clean silicon (Si) substrates and allowed to dry for more than 24 h. The images were taken with a 2 kV acceleration voltage and a 25 pA electron beam current. Energy-dispersive X-ray spectroscopy (EDX) was performed using a detector EDAX Octane Plus (Ametek B.V., Tilburg, Netherlands) coupled to the SEM previously mentioned, for the verification of the presence of Te in the structures. SEM conditions for EDX measurements were a 10 kV acceleration voltage and a 400 pA beam current.

In agreement with TEM microscopy results, spherical TeNPs were observed, see Figure A4. However, in this case, the size distribution is broader than the one found in TEM, and the NPs are bigger; indeed, AV-TeNPs with diameters ranging from 50 to 250 nm can be seen by SEM. This could be related to the time evolution of the nanoparticles, in agreement with the above mentioned evolution of the Z–potential value, as SEM measurements were performed on 3 month old samples.

A subsequent EDX analysis (Figure A5A,B) was carried out in order to identify the elements present in the NPs. Figure A5A shows the X-ray spectra originating from the full area imaged in Figure A4A, as well as that obtained when the electron beam remained stationary on top of the bright NP indicated by a red circle in the same image. The elements detected by the presence of X-ray peaks are shown in the graph. The obtained results confirmed that the NPs contain Te, as well as other elements from the synthesis precursors, in particular, C, O, Ca, Mg, Zn, Mn, K, and Cl can be found in AV [85,86].

The element distribution obtained from the spectra fitting is shown, both for the full image area and for the spot (Figure A5B). As the presence of the Si peak due to the substrate used for sample preparation is not relevant for compositional analysis, the distribution of the elements has been renormalized by removing the Si content and rescaling the rest of the elements to 100% composition. It has to be noticed that due to the heterogeneity of the sample, an accurate quantitative analysis is not possible with EDX (the error bars for the atomic content, not shown in the figure for clarity, are considerably high, mainly for the lighter elements.). However, this approximated quantification allowed us to confirm that Te is mainly located in the NPs, as it can be seen by comparing the obtained amount of Te in the spot and in the full area. The spot corresponds to one of the biggest and brightest particles, and therefore, this indicates that the Te content is higher in this case, suggesting that the core of the NPs is made of Te. The rest of the elements present in the sample could be either incorporated in the NPs or part of the organic coating embedding the nanoparticles.

### Appendix A.5. X-ray Photoelectron Microscopy Analysis

This section contains a discussion of the XPS results for elements other than Te. The Ca 2p core-level spectrum (Figure A6A) was fitted with two spin-orbit 1/2 and 3/2 components located at 350.3 and 346.8 eV, respectively. These binding energies correspond to the presence of calcium carbonate (CaCO_3_) [65]. The Na 1s core-level spectrum (Figure A6B) was fitted with a single component located at 1071.5 eV. Additionally, the Na Auger line KL_23_L_23_ was found to be at 989.7 eV. Both, the binding energy of the Na 1s core level and the position of the Auger line are compatible with the presence of sodium carbonate (NaCO_3_) [65], indicating also that sodium was present in a carbonate form. These compounds are primarily found in aloe vera plants [87,88].

The O 1s core-level spectrum (Figure A6C) was fitted using a minimum of two components located at 530.4 eV (69.6%) and 532.5 eV (30.4%). The most intense contribution at 530.4 eV can be ascribed to the Te oxide [87] in agreement with the high content of Te oxides observed in the Te 3d core-level spectrum. The component at 532.5 eV corresponds to carbon–oxygen bonds in the organic compounds [49,88]; therefore, associated with the aloe vera extract. The binding energy of the oxygen is related to the presence of carbonates in the sample and should be around 530.6–531.5 eV, an energy range close to the first component, ascribed to the Te oxide. The inclusion of an additional component for the fitting was ruled out as the proportion of carbonate is much lower than Te oxide and organic compounds, as was mentioned in the analysis of the wide energy range scan (Figure 4 of the main manuscript).

The C 1s core level spectrum could be fitted with a minimum of 3 components. Nonetheless, the fitting procedure forced the third component at the highest binding energy to avoid a too large full width at half maximum. Thus, this indicated the need to incorporate the fourth component, and the resulting fit is displayed in Figure A6D. The most intense component (59% at 285 eV) corresponds to the presence of either C–C or to C–H bonds in the sp^3^ tetrahedral configuration [89] associated to the organic compounds on the sample. The other components at higher binding energies 286.7 eV (17%) and 288.4 eV (12%) were related to the presence of C-O, C-N, and carbonyl C=O bonds, respectively [48]. Although no evidence of carboxyl or carbonates (both at around 289 eV) was found, a component at lower binding energy (280.3 eV) could be identified. It was inferred that this component corresponds to C atoms weakly bound in the organic compound.

### Appendix A.6. Secondary Ion Mass Spectrometry Imaging

Correlative secondary electron (SE) and elemental imaging were performed on the Zeiss ORION NanoFab configured with a secondary ion mass spectrometer (SIMS), which enables the simultaneous detection of up to 4 atomic and small clusters species. The ORION NanoFab is a multi-ion beam platform (Ga, He, and Ne) that provides high-resolution SE imaging at a 0.5 nm spatial resolution with a 35 keV He^+^ beam and elemental characterization at a 15 nm spatial resolution with a 20 keV Ne beam. More specifically, elemental characterization was based on the mass analysis of secondary ions (SI) produced upon the impact of a sample with an ion beam, neon in this case. SIMS is a highly sensitive surface analytical technique that allows for the detection of all elements, including isotopes, from the periodic table. The combination of these two modalities on one instrument yielded a direct correlative SE and elemental mapping of the exact same area at high spatial resolution. Samples were prepared by depositing a drop of diluted AV-TeNPs on a silicon chip and left to air dry.

Figure A7A,B shows the mass spectra of both positive and negative secondary ions obtained from an AV-TeNPs suspension. The elements detected via SIMS are in good agreement with the SEM-EDX and XPS analysis. The negative mode mass spectrum additionally reveals the presence of CN^-^, characteristic fragments of organic material in SIMS, as well as TeO^-^ and TeO_2_^-^ clusters ions. The latter could be an indicator of the NP chemical composition, but the presence of residual Na_2_TeO_3_ salts in the solution cannot be excluded.

The SIMS simultaneous acquisition of ^16^O^−^, ^12^C^14^N^−^, ^130^Te^−^, and TeO_2_^−^ ion images (Figure A7C–F) was performed on an area containing aggregated NPs, as shown in the SE image obtained with the He beam (Figure A7G, in gray). Interestingly and although easily detectable (Figure A7B), the TeO_2_^−^ ion signal is, on average, five times smaller than that for Te^−^ in these images. CN^−^ and O^−^ signals also fully correlate with Te^−^ and TeO_2_^−^. The co-registration of the SE and Te^-^ SIMS images confirms that the NPs are highly enriched in tellurium, and the low TeO_2_^−^ signal may provide additional evidence, at the nanometer scale, that a large proportion of the Te salt was effectively reduced to elemental Te as NPs. In addition, the detection of CN^−^ in or at the periphery of the NP is in good agreement with the FT-IR spectroscopy results that demonstrate the concomitance of aliphatic amines with the TeNPs.

### Appendix A.7. Determining the Antimicrobial Activity of AV-TeNPs

As can be observed in Figure A8, the presence of different concentrations of AV-TeNPs produced a significant decay in the bacterial proliferation for both MDR *E. coli* (Figure A8A) and MRSA (Figure A8B), an effect that was especially visible for the first bacteria. Besides, no significant difference was found between the concentrations.

The growth curves in Figure A8 were fitted using Huang’s model (Equation (A1)) for logistic growth (fitted curves not presented), Equation (A1). Huang’s model equation.

(A1) $$c_{0}+A-log\;log\;\left(e^{c_{0}}+\left(e^{A}-e^{c_{0}}\right)e^{-\mu \left(t+\frac{1}{4}log\;log\;\frac{1+e^{-4\left(t-\lambda \right)}}{1+e^{4\lambda }}.\;\right)\;}\right),$$

where *A* is the maximum concentration of bacteria obtained in a given environment also known as the carrying capacity, μ is the growth rate, λ is the lag time, $c_{0}$ is the initial concentration, and t is time [90]. This model fits the lag time better than other models (Gompertz, Baranyi, etc.) with similar fits for carrying capacity and growth rate and is promulgated in an FDA tool [90]. The carrying capacity (Figure A9), growth rate (Figure A10), and lag time (Figure A11) for all trials were analyzed. Following the method of Ho et al., we found the mean, the effect size (Hedges’ *g*), and the 95% confidence interval for the 9 experimental measures [91].

To compute the *p*-values, we used a two-sided Mann–Whitney test. While both MDR *E. coli* and MRSA have a *p*-value < 0.01, the effect size for the carrying capacity against MDR *E. coli* was twice that of MRSA (−0.96 ± 0.04 vs. −0.55 ± 0.15). A decreased carrying capacity is a desirable outcome of antibacterial effectiveness, however, there are no strong correlations in the literature that provide a mechanistic description of why carrying capacity decreases with antibacterial effectiveness.

There was no statistically significant difference between the growth rate of the control and the experimental trials on the growth rate for either MDR *E. coli* or MRSA in the presence of AV-TeNPs. Changes in the growth rate are used to indicate antibacterial effectiveness and are commonly correlated to the antibacterial agent affecting membrane formation [92]. While the absence of this effect cannot be ruled out using this analysis, it is less likely the mechanism of action.

AV-TeNPs had little effect and no statistically significant effect against the MRSA lag time. Against MDR *E. coli*, on the other hand, the lag time had a median effect size of 712 ± 103 min for concentrations of 10–75 µg/mL and a *p*-value < 0.05. This combined with the effect size of carrying capacity, the Z-potential of the particles, and the Z-potential of the bacteria, led us to the conclusion that particle–membrane electrostatic interaction is the most significant contribution to the antibacterial effects.

### Appendix A.8. Cell Fixation and SEM Imaging for Bacteria and Human Cells

For bacterial–AV-TeNPs interaction, both bacterial strains (MDR *E. coli* and MRSA) were inoculated into 5 mL of sterile LB media in a 50 mL Falcon conical centrifuge tube and incubated at 37 °C/200 rpm for 24 h. The optical density was then measured at 600 nm (OD600) using a spectrophotometer. The overnight suspension was diluted to a final bacterial concentration of 10^6^ CFU/mL prior to measuring the optical density. A selected 10 µg/mL AV-TeNPs concentration was mixed with LB media and bacterial solution in a 6-well plate with a glass coverslip attached to the bottom. The coverslips were pre-treated with poly-lysine to enhance cell adhesion right before the experiment. The plate was placed inside an incubator for 8 h at 37 °C.

For human cell–AV-TeNPs interactions, HDF and melanoma cells were seeded in a 6-well plate with a glass coverslip (Fisher Brand) attached to the bottom. After an incubation period of 24 h at 37 °C in a humidified incubator with 5% CO_2_, the media was removed and replaced with a fresh one containing a concentration of 50 µg/mL of AV-TeNPs. Cells were cultured for another 24 h at the same conditions.

After their corresponding incubation/culture periods, the coverslips were fixed with a primary fixative solution containing 2.5% glutaraldehyde and 0.1 M sodium cacodylate buffer solution for 1 h. Subsequently, the fixative solution was exchanged for 0.1 M sodium cacodylate buffer, and the coverslips were washed 3 times for 10 min. Post-fixation was done using a 1% osmium tetroxide (OsO_4_) solution in the buffer for 1 h. Subsequently, the coverslips were washed three times with buffer, and dehydration was progressively achieved by soaking in 35, 50, 70, 80, 95, and 100% ethanol (with a three time soaking for the 100% ethanol). Finally, the coverslips were dried using a liquid CO_2_-ethanol exchange in a SAMDRI^®^-PVT-3D critical point dryer. The coverslips were mounted on SEM stubs with carbon adhesive tabs (Electron Microscopy Sciences) after treatment with liquid graphite, and then sputter-coated with a thin layer of platinum using a Cressington 208HR High-Resolution Sputter Coater. Images were taken with a Hitachi S-4800 SEM (Hitachi Tokyo, Japan).

SEM micrographs of control MDR *E. coli* and MRSA (Figure A12A,C) and bacteria after treatment with a concentration of 10 μg/mL of AV-TeNPs (Figure A12B,D) are shown. The characterization indicates that the treatment with the TeNPs induced changes on both bacterial strains. Disruption of the outer cell membrane and cell lysis was seen after the treatment with the NPs. Therefore, evident cell damage was observed, with an abundant presence of holes and cracks all over the cell membrane, and bacterial deformation and collapse. Cell membrane damage is commonly found to be a consequence of increased ROS production. From the SEM images of the bacteria, it is possible to observe that membrane damage occurred and that there was attachment of nanoparticles to bacteria, but the exact mechanism of how the damage occurred cannot be identified at this time.

SEM micrographs were also obtained of HDF and melanoma cells with no AV-TeNP treatment (Figure A13A,C) and after treatment at a concentration of 50 μg/mL of AV-TeNPs (Figure A13, respectively. As it can be seen, HDF cells were able to successfully proliferate in the presence of the Te-based nanostructures, with no apparent disruption or alteration of a membrane or average growth. On the other hand, the presence of the AV-TeNPs caused the presence of bubbles and membrane disruption within the melanoma cell population; characteristic morphologies found in cells that die due to apoptotic mechanisms, which can be ascribed to an excess of ROS (see Figure 6 in the main manuscript).
